# Molecular epidemiological characterization of human bocavirus (HBoV) in acute respiratory infection (ARI) patients in Yucheng, China

**DOI:** 10.3389/fpubh.2025.1548907

**Published:** 2025-04-07

**Authors:** Qi Wen, Ronghua Yang, Qin Luo, Qiangqiang Shi, Ze Chen, Chen Gao, Haijun Du, Guoyong Mei, Shuying Li, QinQin Song, Jun Han

**Affiliations:** ^1^National Key Laboratory of Intelligent Tracking and Forecasting for Infectious Diseases, National Institute for Viral Disease Control and Prevention, Chinese Center for Disease Control and Prevention, Beijing, China; ^2^Hebei Key Laboratory for Chronic Disease, College of Basic Medicine, North China University of Science and Technology, Tangshan, China; ^3^Yucheng Center for Disease Control and Prevention, Shangqiu, China

**Keywords:** acute respiratory infection, human bocavirus, epidemiology, genetic characteristics, amino acid mutation, clinical characteristics

## Abstract

**Objective:**

To investigate the epidemiological characteristics and genetic profile of human bocavirus (HBoV) among patients with acute respiratory infection (ARI) in Yucheng, Henan, China.

**Methods:**

A total of 1,153 throat swabs were collected from ARI patients between March and June 2023. These samples were tested for 18 pathogens using quantitative chain reaction (qPCR). The VP1 gene were amplified and sequenced from HBoV-positive samples. The viral genetic characterization was analyzed by comparing the HBoV1 sequences with reference sequences.

**Results:**

Of the 1,153 samples, the detection rate of HBoV positive was 6.85%, primarily detected in May 2023. The majority of HBoV positive cases were found in children under 5 years old. Clinical manifestations in HBoV-positive patients predominantly were fever and cough, with a clinical diagnosis of lower respiratory tract infection. Other viruses were also detected in 20% of HBoV-positive samples. All HBoV sequences identified in this study belonged to HBoV1, with the Ib sublineage being the predominant strain circulating in Yucheng during March–June 2023.

**Conclusion:**

After the implementation of the optimized COVID-19 prevention and control strategy in December 2022, HBoV infection was prevalent in Yucheng, Henan in May-June 2023, mainly among children younger than 5 years old, especially those under 2 years old. The Ib sublineage of HBoV was the dominant circulating strain in Yucheng, Henan during March–June 2023.

## 1 Introduction

Acute respiratory infection (ARI) is a leading cause of morbidity and mortality globally, especially in low- and middle-income countries, where ARI-associated deaths accounted for 83% of child mortality before the COVID-19 pandemic (1).Among them, viruses are the most important pathogens causing ARI (2), and according to the World Health Organization(WHO), there have been several global outbreaks of respiratory viruses, such as influenza A viruses, Middle East respiratory syndrome coronavirus (MERS-CoV), and SARS-CoV-2 in 2019, which have resulted in a huge disease burden for humans (3, 4). Therefore, understanding the epidemiological characteristics of viruses as well as their genetic evolutionary features is important for human prevention and control of virus transmission and treatment of viral infections.

Human bocavirus (HBoV) is recognized as an important cause of acute respiratory tract infections (5), which was first identified in 2005 from nasopharyngeal swabs of children with respiratory disease in Sweden (6), and four genotypes are known (HBoV1-4) (7, 8). HBoV is a single-stranded, linear, non-envelope DNA virus with a 20-hedral structure, belonging to the genus Bocavirus in Parvoviridae (9–11). Globally, the prevalence of HBoV is estimated at 6.3% in respiratory infections and 5.9% in gastrointestinal infections (12). Children are more susceptible to the infection than adults and older adults, especially children under 5 years of age (13). HBoV infection mainly cause clinical manifestations such as cough, runny nose, shortness of breath, and dyspnea. The medical diagnoses typically include pneumonia, bronchitis, rhinitis, and tonsillitis (13–15). However, HBoV infection can lead to severe respiratory diseases such as severe pneumonia and acute respiratory failure (16), and the rate of HBoV co-infection with other pathogens is also high (11, 16). Lacking of effective viral culture methods and animal infection models for HBoV infection, there is a limited number of molecular epidemiologic studies of HBoV (17, 18).

After the implementation of the optimized COVID-19 prevention and control strategy in December 2022, there was an increase in the number of ARI cases in Yucheng, China, with HBoV detection at the top of the list. Due to the lack of a well-established molecular epidemiological detection system for HBoV and the limited research on its genotypic distribution and genetic characterization in China, this study aimed to enrich the epidemiological data of HBoV in ARI as well as the molecular evolutionary characterization of HBoV through in-depth research on the molecular epidemiology of HBoV, providing new insights into the prevention and control of HBoV.

## 2 Materials and methods

### 2.1 Study subjects

A total of 1,153 throat swabs of ARI patients from March to June 2023 were collected by the sentinel hospital in Yucheng, China. Basic demographic information, sampling date, diagnosis, clinical symptoms, and biochemical test results were also collected. Inclusion criteria for ARI patients: (1) Acute manifestations of infection (at least one of the following): fever; elevated or decreased white blood cells; chills; abnormally low body temperature; (2) Respiratory clinical manifestations (at least one of the following): upper respiratory tract symptoms such as pharyngeal discomfort, dryness or soreness of the pharynx; nasal congestion, runny nose; obvious congestion and edema of the nose, pharynx, and larynx; cough, sputum, shortness of breath, chest pain, and abnormal respiratory sounds on auscultation. Patients who have used immunosuppressive or antiviral drugs in the last one month and who have a combination of serious underlying diseases are not collected (19, 20). After collection, the throat swabs were immediately stored at −80°C and transported in dry ice throughout the cold chain to the laboratory of the Virus Resource Center, National Institute for Viral Disease Control and Prevention, Chinese Center for Disease Control and Prevention for further testing.

### 2.2 Nucleic acid extraction and pathogen detection

Nucleic acids were extracted from throat swabs using a fully automated nucleic acid extractor (Art. No.: NP968, Xi'an Tianlong Technology Co., Ltd., Xi'an, China) and the corresponding Magnetic Bead Method Viral DNA/RNA Rapid Extraction or Purification Kit (Art. No.: NP968, Xi'an Tianlong Technology Co., Ltd.), following the manufacturer's instructions. Real-time fluorescence quantitative PCR kit GoldStar Probe One Step RTq-PCR Kit (Art. No.: CW22075S, Jiangsu Kangwei Century Biotechnology Co., Ltd., Jiangsu, China) and GoldStar Probe Mixture (Art. No.: CW09322M, Jiangsu Kangwei Century Biotechnology Co., Ltd., Jiangsu, China) were performed to detect 18 respiratory pathogens according to the instructions and screened positive samples for pathogens. (The experiment is successful if both negative and positive controls are normal.)The pathogens tested for included: human rhinovirus (HRV), respiratory syncytial virus (RSV), human metapneumovirus (HMPV), influenza viruses A and B (FluA and FluB), human parainfluenza virus types 1-3 (HPIV1-3), and human coronaviruses (OC43, NL63, 229E, and HKU1), adenovirus (AdV), human bocavirus (HBoV), enterovirus (EV), mycoplasmal pneumonia (MP), chlamydia pneumoniae (CP), severe acute respiratory syndrome coronavirus 2 (SARS-CoV-2). Primers and probes for these 18 respiratory pathogens were synthesized by Sangon Bioengineering Co., Ltd. (Shanghai, China).

### 2.3 HBoV VP1 gene amplification and sequence determination

The VP1 gene was amplified using nested PCR on HBoV-positive pharyngeal swab samples with primer sequences referenced in Tables 1, 2 (21). Nested PCR was performed using the kit 2 × Es Taq Master Mix (Dye) (Art. No.: CW0690M, Jiangsu Kangwei Century Biotechnology Co., Ltd., Jiangsu, China). The first round of PCR reaction conditions were: 94°C for 4 min, 94°C for 30 s, 45°C for 45 s, 72°C for 1 min 40 s, amplification of 35 cycles, the last 72°C extension for 10 min, the products were stored at 4°C. The second round of PCR reaction was modeled on the first round of PCR products, and the amplification conditions were: 94°C for 4 min, 94°C for 30 s, 45°C for 40 s, 72°C for 1 min 10 s, amplification for 35 cycles, and finally 72°C extension for 8min, and the products were stored at 4°C. After the nested PCR, the products were identified by 1.5% agarose gel electrophoresis, and the positive PCR products were sent to Tsingke Bioengineering Co., Ltd. (Beijing, China) for bidirectional sequencing.

**Table 1:** First round amplification primers of nest PCR of HBoV VP1 gene.

**Primer**	**Sequence (5′-3′)**	**Target (nt)**	**Size (bp)**
FA-F	TGGCAGACAACTCATCACAGGA	2,491–2,512	1,000
FA-R	TGTCTGAAAAGTGAGAACCTCCG	3,574–3,596	
FB-F	TTTAAAATAAAGCGCGCCGTGG	3,308–3,329	953
FB-R	GCTTGTTGGTTTTGAGTATGGC	4,309–4,330	
FC-F	TCGCAGATCTTGATGGAAATGC	4,038–4,059	1,031
FC-R	TGTACAACAACAACACATTAAAAGA	5,275–5,299	

F, Forward primer; R, Reverse primer.

**Table 2:** Second round amplification primers of nest PCR of HBoV VP1 gene.

**Primer**	**Sequence (5′-3′)**	**Target (nt)**	**Size (bp)**
SA-F	ACACAGTGGGGAGAGAGGCT	2,535–2,554	1,000
SA-R	CCTCCTCCAATACTTCCTGTTCC	3,512–3,534	
SB-F	TCTGGGAAATAAAGAGAGAGCC	3,337–3,358	953
SB-R	CTGCTGTGCTTCCGTTTTGTCT	4,268–4,289	
SC-F	TTGAAAACAGTGACCACCAAGT	4,116–4,137	1,031
SC-R	TACAGTCACCCTTCACTTTCCG	5,125–5,146	

F, Forward primer; R, Reverse primer.

SeqMan 7.1.0 was used to organize and splice the sequenced sequences. The spliced sequences were subjected to BLAST on NCBI for preliminary determination of serotypes. The original HBoV strains st1 and st2 and other related reference sequences were downloaded from NCBI GenBank for subsequent analysis. Sequence comparison was performed using MEGA 6.0 [Maximum Likelihood (ML) was used, and the bootstrap value was set to 1,000] to construct phylogenetic trees, analyze amino acid variant sites and calculate genetic distances. The evolutionary tree was beautified using ITOL (http://itol.embl.de/). Evolutionary homology analysis was performed using MegAlign software. Amino acid key site analysis results were visualized using the WebLogo online tool (http://weblogo.berkeley.edu/logo.cgi).

### 2.4 Glycosylation sites of VP1 protein

The N/O-glycosylation site of HBoV1 VP1 protein was, respectively, predicted using the NetNGlyc 1.0 and NetOGlyc 4.0 online server under the default conditions (https://services.healthtech.dtu.dk/). A threshold value >0.5 was used, and the presence of the sequence Asn-X-Ser/Thr (X is any amino acid other than proline) indicated a potential N-glycosylation site, the presence Ser, Thr or Hypindicated a potential O-glycosylation site.

### 2.5 Statistical analysis

**Figure 1:**
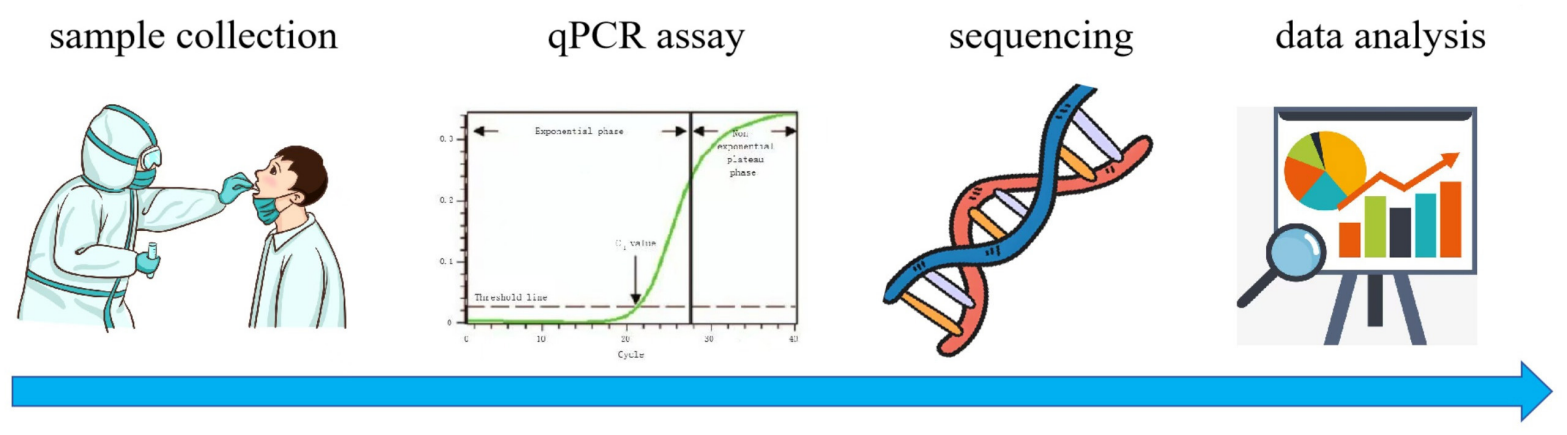
Flowchart of the experiment. Pharyngeal swab samples from ARI patients in Yucheng were collected and screened for HBoV-positive samples using qPCR to analyze the epidemiological and molecular evolutionary characteristics of HBoV patients.

Microsoft Excel 2019 and SPSS 27.0 software were used for data organization and statistical analysis. Graphs were plotted and embellished using GraphPad Prism 10.1.2 software. Skewed distribution measures were expressed as median (interquartile spacing), enumeration data were expressed as number of cases or percentages, and comparisons of differences in rates (or constitutive ratios) between groups for enumeration data variables were performed using the χ^2^ test or Fisher's exact probability method. Statistics were performed using a two-sided test with a test level of α = 0.05, and a statistically significant difference of *P* < 0.05 (Figure 1).

## 3 Result

### 3.1 HBoV detection of ARI patients' samples

**Figure 2:**
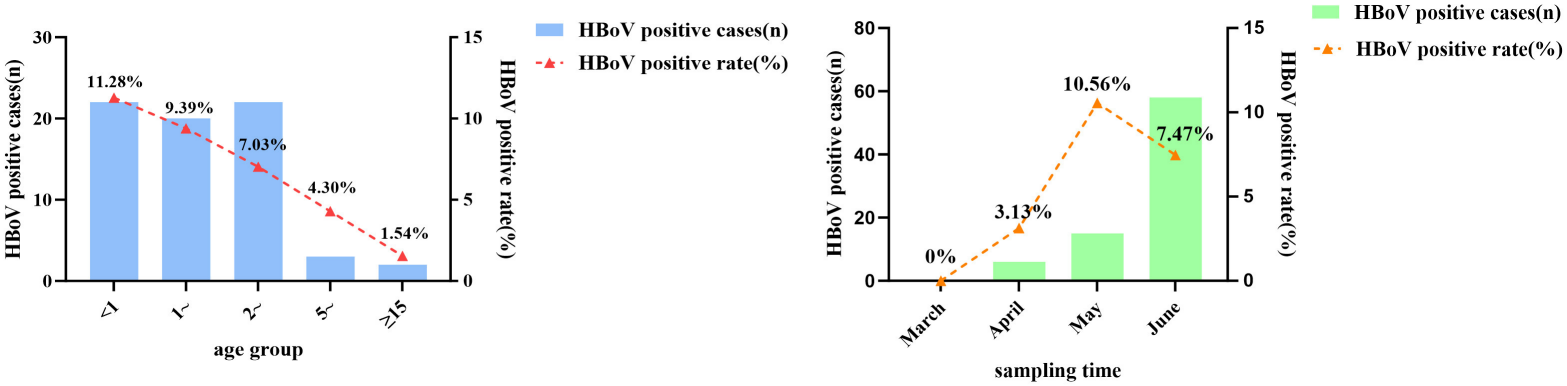
Positivity of HBoV-positive patients in terms of age group and sampling time.

Out of the 1,153 swabs' samples tested, 79 were positive for HBoV, giving an overall detection rate of 6.85%. Among males, the detection rate was 7.62% (48/630), while for females, it was 5.93% (31/523), resulting in a male to female ratio of 1.55:1. Although males had a higher detection rate, the difference between genders was not statistically significant (*P* = 0.258). The age range of HBoV positive patients was 0.1–32 years (Median age is 3.3 years.). Among the age groups, the highest detection rate was 11.28% (22/195) in patients under 1 year old;: followed by group aged over 1 and under 2 years old, with a detection rate of 9.39% (20/213), and the difference in detection rates among different age groups was statistically significant (*P* = 0.002). In terms of sampling time, the highest detection rate was 10.56% (15/142) in May, followed by 7.47% (58/776) in June, and the difference between detection rate of different sampling times was statistically significant (*P* = 0.008) (Figure 2, Table 3).

**Table 3:** Epidemiological characteristics of HBoV positive patients.

**Clinical information**	**Total cases**	**Positive cases**	**Positivity rate (%)**	***P-*value**
Gender				0.258
Male	630	48	7.62	
Female	523	31	5.93	
Age (years old)				0.002
< 1	195	22	11.28	
1~	213	20	9.39	
2~	313	22	7.03	
5~	302	13	4.30	
≥15	130	2	1.54	
Sampling time				0.008
March 2023	43	0	0	
April 2023	192	6	3.13	
May 2023	142	15	10.56	
June 2023	776	58	7.47	

Distribution of HBoV-positive patients by sex, age and sampling time.

### 3.2 The clinical manifestations of HBoV infections

**Figure 3:**
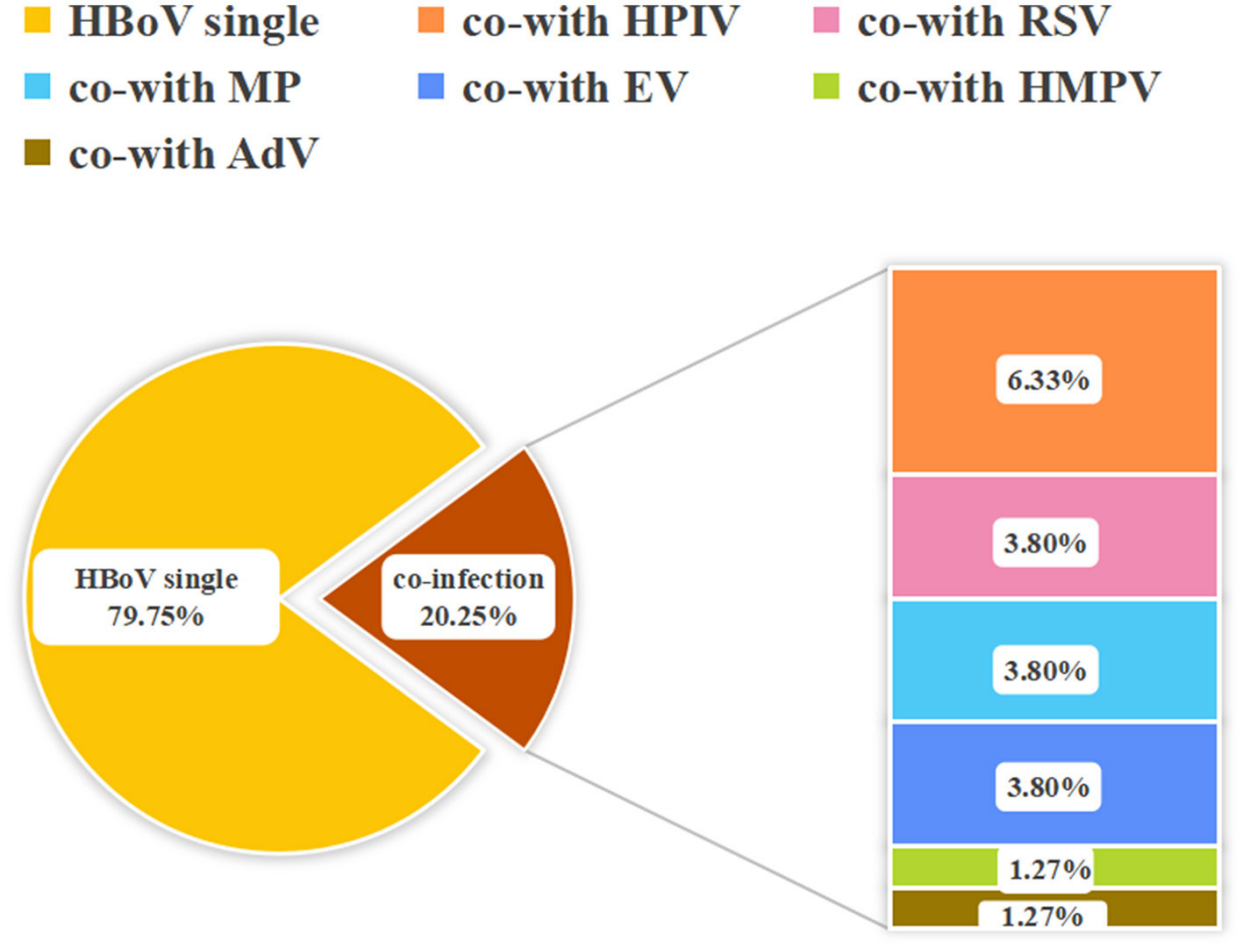
Proportion of other pathogens in HBoV co-infections.

**Figure 4:**
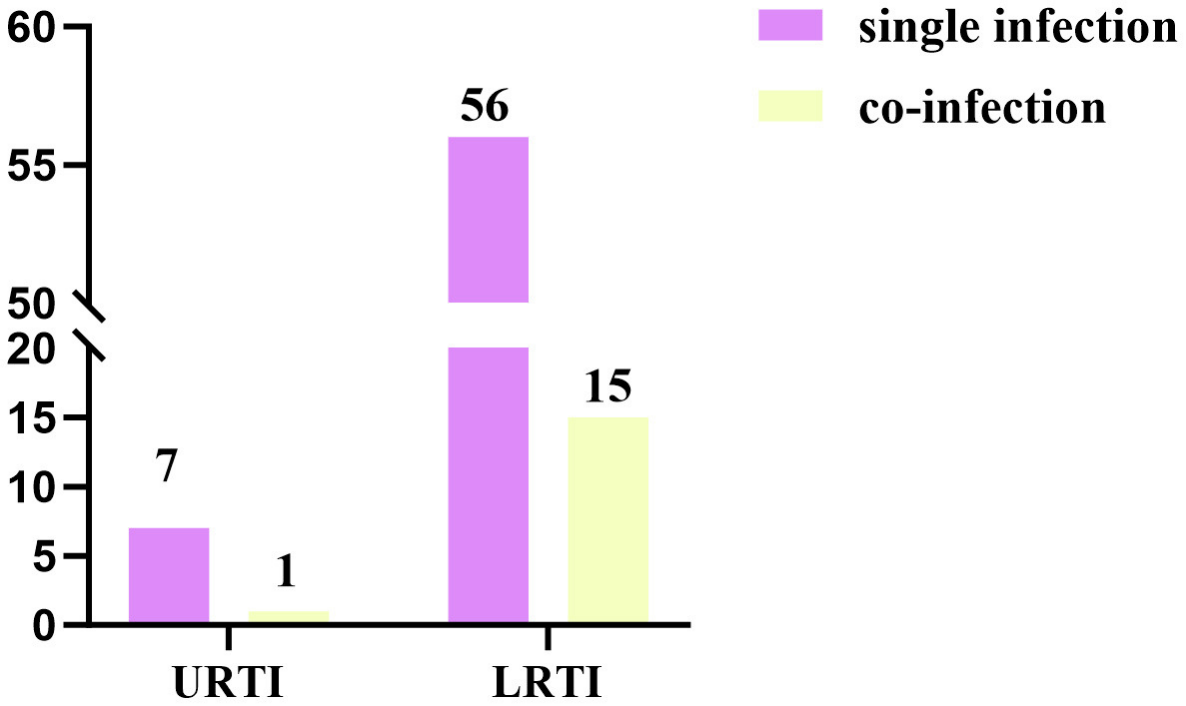
Distribution of the number of HBoV-positive cases between upper and lower respiratory tract infections in single infections and co-infections.

Among the 79 HBoV-positive patients, 63 samples (79.7%) were single HBoV positive, and 16 samples (20.3%) were positive for other pathogens in addition to HBoV. All of the co-infections were dual infections, with the most common being co-infection of HBoV and HPIV (6.33%, 5/79) (Figure 3). In HBoV-positive patients, the most common clinical manifestations were fever and cough, and the clinical diagnosis was predominantly bronchopneumonia in lower respiratory tract infection (LRTI) (70/79, 88.6%). In co-infected cases, the clinical diagnosis was mainly bronchopneumonia (15/16, 93.8%), except for one patient who developed myocardial damage (Figure 4).

### 3.3 Analysis of genotype of HBoV

After the VP1 gene sequences were amplified by nested PCR from HBoV-positive samples, 15 HBoV VP1 gene sequences were finally obtained. To analyze phylogenetic characteristic of above sequences of HBoV, a dataset containing 52 representative sequences of HBoV1 VP1 from different regions and time on NCBI GenBank was constructed for the subsequent analyses by using the MEGA6.0 software to perform sequence comparison (Supplementary Table 1).

The VP1 gene sequence homology firstly was analyzed among the 15 HBoV1 strains in this study. The results showed that their nucleotide homology ranged from 99.6% to 100.00%, and their amino acid homology ranged from 99.7% to 100.00%. Compared with the VP1 gene sequences of the reference HBoV1 strains st1 (DQ000495) and st2 (DQ000496), the nucleotide homology was 99.6%−100.0% and 99.6%−100.00%, and the amino acid homology was 99.6%−100.0% and 99.7%−100.00%, respectively, and this data indicated that the HBoV1 VP1 gene is highly conserved.

**Figure 5:**
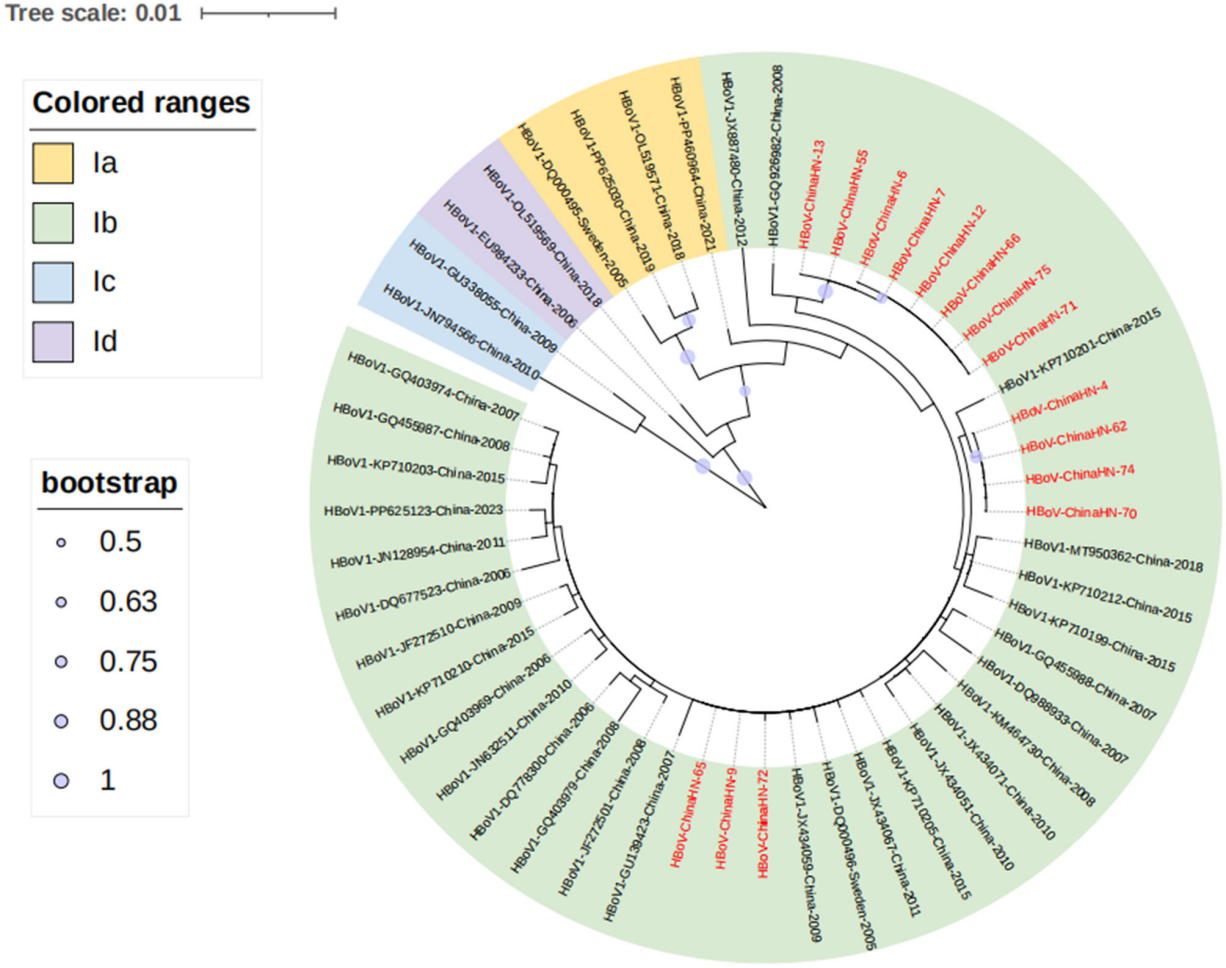
Phylogenetic analysis of the VP1 gene of 15 Yucheng strains. HBoV1 can be categorized into four branches, Ia, Ib, Ic and Id, and all 15 Yucheng strains obtained in this study were distributed in the Ib branch, and the sequences obtained in this study are shown in *red*.

To understand the evolutionary characteristics of the HBoV in this study, the VP1 sequence was subjected to phylogenetic tree analysis with 52 sequences from different countries, regions and periods using MEGA6.0 software. Based on previous research (21), HBoV1 VP1 gene sequence can be categorized into four branches, namely, Ia, Ib, Ic, and Id. The representative sequences of the four branches of HBoV were also chosen to construct the phylogenetic tree, including the original strain st1 (DQ000495) of the Ia branch (21, 22), the original strain st2 (DQ000496) of the Ib branch (21, 22), and two branches of Ic represented by the Guangdong strain (JN794566) (GU338055) (21, 22), and the Id branch represented by one strain of Taiwan (EU984233) (21), China. The prevalent strains obtained in this study were all located in the Ib branch from the phylogenetic tree, thus the Ib branch was the dominant prevalent strain, which was clustered with the dominant strain of HBoV1 in China. The genetic distances between the 15 Yucheng strains and other prevalent strains in several regions of China in 2006–2023 were 0.000–0.020. Among the fifteen Yucheng strains, eight Yucheng strains (HBoV-ChinaHN-6/7/12/13/55/66/71/75) were more closely related to the 2008 Guangdong strain (GQ926982) (99.6%-99.7%concordance).The four Yucheng strains (HBoV-ChinaHN-4/62/70/74) were more closely related to the 2018 Guangdong strain (MT950362) (99.8% concordance). The three Yucheng strains (HBoV-ChinaHN-9/65/72) were more closely related to the 2009 Chongqing strain (JX434059) (99.9%−100.0% concordance). The fifteen HBoV1 strains from Yucheng identified in this study did not show significant geographic and temporal clustering, suggesting that the Yucheng HBoV1 strains obtained in this study may have originated from different transmission chains (Figure 5).

### 3.4 Analysis of nucleotide and amino acid variants in the VP1 gene

The 15 HBoV VP1 gene sequences obtained in this study showed high conservation compared to the original strains st1 and st2. No nucleotide deletions or insertions were found in these sequences. However, 26 nucleotide variant sites and five amino acid variant sites were identified in these sequences. Specifically, compared with the original strain st2 of Ib branch, amino acid N474S substitution was found in all 15 Yucheng strains, amino acid L40S substitution was found in eight strains and amino acid G96E substitution appeared only in one strain. The HBoV1 VP1 gene, which encodes a protein of 671 amino acids (aa), has several regions crucial to viral antigenicity, including the VP1u region (1-129aa), the N-terminal region of VP3 (130-165aa), and the region of βG-βH insertion sequence (374-576aa). All three amino acid variant sites in this study were located therein. In addition, one amino acid variant is located in VP1u region but is not a critical amino acid site for maintaining phospholipase A2(PLA2) activity (Pro 21, His 41, Asp 42, or Asp 63) (23) (Table 4).

**Table 4:** Comparison of amino acid variation sites of VP1 gene of HBoV strains in Yucheng with st2 of the original strain.

**HBoV**	**Amino acid sites**		
	**40**	**96**	**474**
HBoV-DQ000496-Sweden-2005	L	G	N
HBoV-ChinaHN-4	.	.	S
HBoV-ChinaHN-6	S	.	S
HBoV-ChinaHN-7	S	.	S
HBoV-ChinaHN-9	.	.	S
HBoV-ChinaHN-12	S	.	S
HBoV-ChinaHN-13	S	.	S
HBoV-ChinaHN-55	S	.	S
HBoV-ChinaHN-62	.	.	S
HBoV-ChianHN-65	.	.	S
HBoV-ChinaHN-66	S	.	S
HBoV-ChinaHN-70	.	.	S
HBoV-ChinaHN-71	S	.	S
HBoV-ChinaHN-72	.	E	S
HBoV-ChinaHN-74	.	.	S
HBoV-ChinaHN-75	S	.	S

The VP1 gene sequence of HBoV original strain st2 was used as the reference sequence. The amino acid sites in the Yucheng strain that is same those in the original strain st2 is represented by a dot.

**Figure 6:**
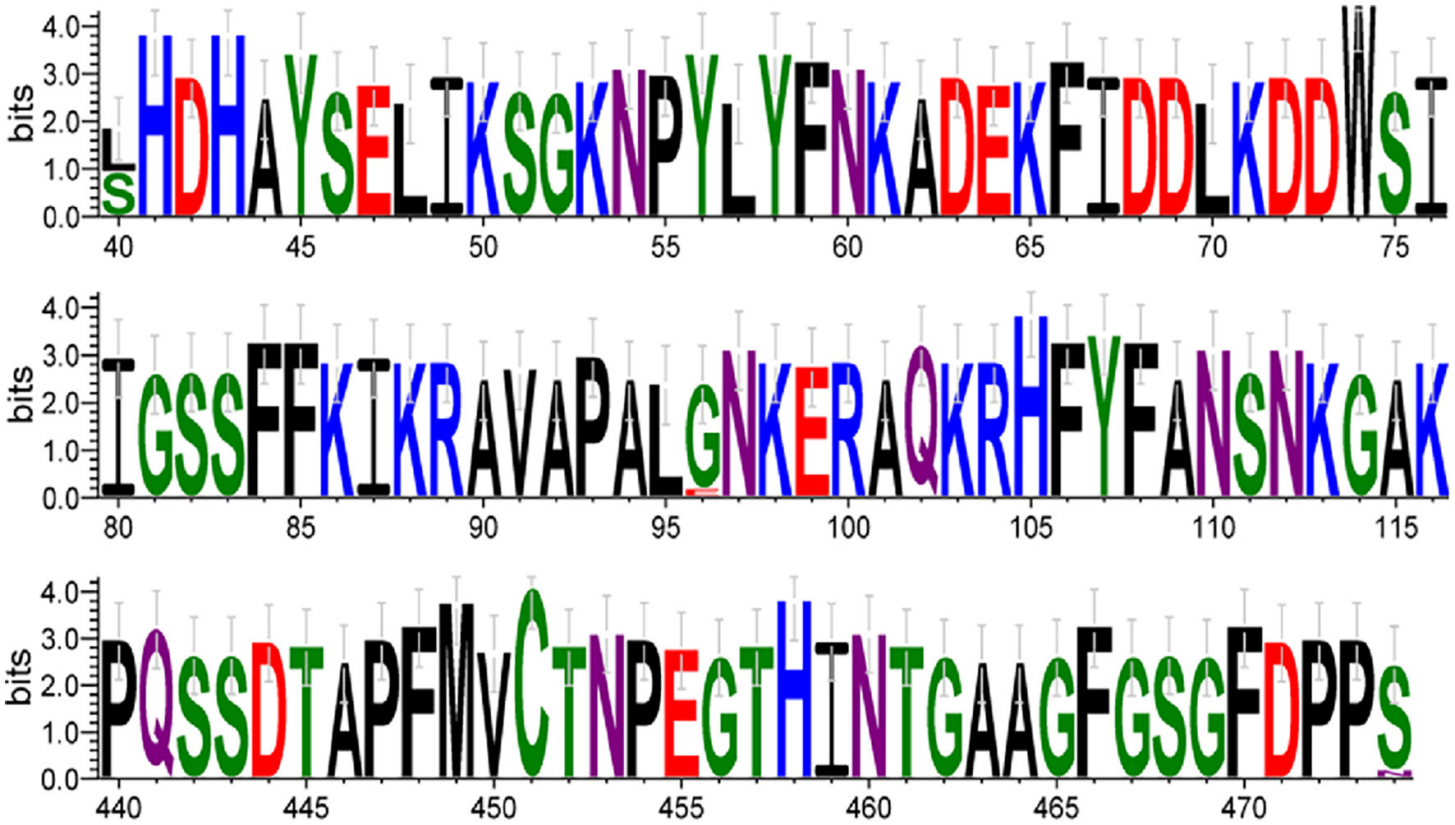
The font size of each amino acid is proportional to the percentage of conservation at each position. The numbers at the bottom indicate the amino acid positions. Amino acids are colored according to their chemical properties: polar amino acids (G, S, T, Y, C) are shown in *green*, neutral (Q, N) in *purple*, basic (K, R, H) in *blue*, acidic (D, E) in *red*, and hydrophobic (A, V, L, I, P, W, F, M) amino acids in *black*.

Key amino acid variant sites were visualized and analyzed, showing amino acids substitution appeared at the same position at N40, N96, and N474, respectively (Figure 6).

### 3.5 Prediction of VP1 protein glycosylation sites

The results showed that three potential N-glycosylation sites (N296, N339, and N407) existed in 15 HBoV1 VP1 proteins prevalent in Yucheng, China during March and June 2023, which were consistent with the potential N-glycosylation sites of st2 VP1 protein of the original HBoV1 strain.

Compared with the O-glycosylation sites of the original strain st2 (N46, N111, N118, N121, N127, N128, N131, N133, N142, N149, N150, N154, N156, N410, N413, N421, N442), there were three more glycosylation sites (N443, N468, N491) in six Yucheng strain (HBoV-ChinaHN-4/9/62/65/70/74). There were three more glycosylation sites (N468, N480, N491) in eight Yucheng strains (HBoV-ChinaHN-6/7/12/13/55/66/71/75). There were two more glycosylation sites (N468, N491) and three less glycosylation sites (N413, N421, N442) in one Yucheng strain (HBoV-ChinaHN-72).

## 4 Discussion

Human bocavirus (HBoV) is strongly associated with acute respiratory infection (ARI) (22), and during the COVID-19 pandemic, pathogens other than SARS-CoV-2 were in a low-prevalence state due to the implementation of the Non-Pharmaceutical Interventions (NPIs) (24), whereas a resurgence of respiratory pathogens has been observed in several national geographies after the relaxation of NPIs (25, 26). In this study, HBoV detection ranked first, higher than other studies in Kunming, Guangzhou and Suzhou regions of China (27–29). According to Zhao et al. (30), an increase of HBoV was observed in ARI patients after the of NPIs, and the results of the present study also verified this finding.

HBoV can cause severe acute respiratory infection as an isolated pathogen and can be latently present and persistent (14). In the present study, most of the HBoV-positive patients were diagnosed with bronchopneumonia, with the highest percentage in children under five years of age, and it has been shown that children infected with HBoV are more susceptible to pneumonia and wheezing (31), and that in immunocompromised populations, such as infants and young children, HBoV infections pose a more severe burden of disease (32). In the present study, HBoV infection showed a decreasing trend with increasing age group, with a high prevalence in children and a lower prevalence in adults and older adults, similar to the findings of Kim et al. (33). According to seroepidemiologic studies of HBoV, humoral and cellular immune responses against HBoV are prevalent in adolescents and adults, and it is likely that all children have been exposed with HBoV by the age of six years (34, 35).

Co-infections of HBoV are very common and mainly dominated by dual infections (36–38). The most common viruses in co-infections were RSV, HRV, and HPIV (12, 39). The low rate of co-infections in this study may be related to the limited sampling time in this study (from March to June), and the fact that many respiratory viruses are more prevalent in the winter season (40, 41). The clinical diagnosis of patients with co-infections was overwhelmingly bronchopneumonia, in addition to this, myocardial damage was found in a male, aged nine months, who was also infected with RSV, and it has been shown that clinical symptoms are more severe in patients with HBoV-RSV co-infections (42). In addition to this, it has been observed in other studies that children patients with combined HBoV infections have longer hospital stays and may develop serious complications such as pneumothorax, encephalitis, and mediastinal emphysema (43). Patients with co-infections may then need to be treated with antibiotics, intensive respiratory support or even mechanical ventilation.

There are geographical differences in HBoV infection, and some studies have indicated that climate and environment may be factors influencing virus transmission (29). Yucheng is located in the north of China, where the climate is mainly temperate climate, and HBoV detection were higher in the spring, a result similar to that of studies in Japan and Korea (44, 45). The high detection rate in this study may also be due to the temperature and humidity in May being suitable for the survival and transmission of the virus. In southern China, the climate is mainly subtropical, and the peak of HBoV epidemics mainly in the summer (28, 29, 39); but in Rome (46), Australia (47) and the United Kingdom (48), the detection rate is higher in the winter. In addition to this, China experienced a high prevalence of influenza A in March 2023 (49), and people strengthened their personal protection, which reduced the spread of the virus, while influenza A was in a low-prevalence state in May, and interpersonal communication increased, which facilitated the spread of the virus. However, many viruses are predominantly endemic in fall and winter, such as RSV and HRV (50, 51), suggesting that attention should be paid to the prevalence of HBoV in the alternating spring and summer time.

Based on the phylogenetic analysis of the HBoV VP1 gene, the results indicated that all Yucheng strains obtained in this study belonged to the Ib branch of HBoV1, which was the same as the dominant strains in several Chinese provinces from 2006 to 2023. The three amino acid site variants found at positions 40, 96, and 474 were also reported in a previous study (22), indicating that the HBoV1 VP1 gene sequences of the prevalent HBoV1 strains in China, including Yucheng, Henan, are relatively conserved, and the trend of viral evolution is relatively stable, which is also in line with the characteristic of DNA viruses that are slower to evolve in relation to RNA viruses. Moreover, the ability to retain these amino acid sites during strain epidemics suggests that these amino acid sites may provide a potential advantage for HBoV transmission.

Currently, there is no specific antiviral drug for the treatment of HBoV (12, 52), and the prevention and control of HBoV should be strengthened. Firstly, by strengthening personal hygiene habits (washing hands diligently, wearing masks correctly, etc.); for infants, young children and immunocompromised people, they should reduce their visits to densely populated or poorly ventilated places (e.g., shopping malls, hospitals, etc.). Secondly, for public places such as day-care centers and other venues, windows should be regularly opened for ventilation, public items should be disinfected regularly; and public health institutions should carry out publicity on the prevention of HBoV infection. Thirdly, sentinel hospitals in each region should strengthen the monitoring of HBoV (especially during the high HBoV season). Fourthly, in terms of prevention and treatment, virus-like particle (VLP) vaccines and antiviral target drugs are the main research directions in the future (12). Fifthly, whole genome sequencing technology can provide a better understanding of the evolutionary features of HBoV or the emergence of new sublineages (53).

By analyzing the epidemiological and genetic characteristics of HBoV among ARI patients in Yucheng, this study added some data on HBoV in this region after the COVID-19 pandemic, and provided a scientific basis for the subsequent understanding of the epidemiological characteristics and pathogenicity of HBoV in other regions of China. However, there are some limitations in this study, as the ARI data collected were concentrated in time and no bacterial testing was performed. We will continue to collect ARI data for long-term monitoring.

## 5 Conclusion

In summary, HBoV was the number one pathogen in Yucheng, China, from March to June 2023. HBoV mainly occurs to infect children under five years of age, and the clinical symptoms were mainly fever and cough. The Ib branch of HBoV1was the main prevalent strain in Yucheng.

## Data Availability

The datasets presented in this study can be found in online repositories. The names of the repository/repositories and accession number(s) can be found in the article/Supplementary material.
