# Supplementary material for: Molecular epidemiological characterization of human bocavirus (HBoV) in acute respiratory infection (ARI) patients in Yucheng, China
Source: Front Public Health. 2025 Apr 7;13:1548907. doi: 10.3389/fpubh.2025.1548907 (PMC12009866; doi:10.3389/fpubh.2025.1548907)
Supplement: Supplementary file 1 [file Table_1.docx]

| HBoV sequences | GenBank accession number |
| --- | --- |
| HBoV-ChinaHN-4 | PV232614 |
| HBoV-ChinaHN-6 | PV232615 |
| HBoV-ChinaHN-7 | PV232616 |
| HBoV-ChinaHN-9 | PV232617 |
| HBoV-ChinaHN-12 | PV232618 |
| HBoV-ChinaHN-13 | PV232619 |
| HBoV-ChinaHN-55 | PV232620 |
| HBoV-ChinaHN-62 | PV232621 |
| HBoV-ChinaHN-65 | PV232622 |
| HBoV-ChinaHN-66 | PV232623 |
| HBoV-ChinaHN-70 | PV232624 |
| HBoV-ChinaHN-71 | PV232625 |
| HBoV-ChinaHN-72 | PV232626 |
| HBoV-ChinaHN-74 | PV232627 |
| HBoV-ChinaHN-75 | PV232628 |

Supplementary Table 1 GenBank accession number of the HBoV sequence
